# Association of brain–autonomic activities and task accuracy under cognitive load: a pilot study using electroencephalogram, autonomic activity measurements, and arousal level estimated by machine learning

**DOI:** 10.3389/fnhum.2024.1272121

**Published:** 2024-02-29

**Authors:** Naoya Sazuka, Koki Katsumata, Yota Komoriya, Takeyuki Oba, Hideki Ohira

**Affiliations:** ^1^Human Technology Research and Development Department, Application Technology Research and Development Division, Technology Development Laboratories, Sony Corporation, Tokyo, Japan; ^2^Department of Cognitive and Psychological Sciences, School of Informatics, Nagoya University, Nagoya, Japan

**Keywords:** cognitive load, electroencephalogram (EEG), heart rate variability, skin conductance response, machine learning, infra-slow fluctuations of alpha power

## Abstract

The total amount of mental activity applied to working memory at a given point in time is called cognitive load, which is an important factor in various activities in daily life. We have proposed new feature quantities that reflect the time-series changes in the power of typical frequency bands in electroencephalogram (EEG) for use in examining the relationship between brain activity and behavior under cognitive load. We also measured heart rate variability (HRV) and spontaneous skin conductance responses (SCR) to examine functional associations among brain activity, autonomic activity, and behavior under cognitive load. Additionally, we applied our machine learning model previously developed using EEG to the estimation of arousal level to interpret the brain–autonomic–behavior functional association under cognitive load. Experimental data from 12 healthy undergraduate students showed that participants with higher levels of infra-slow fluctuations of alpha power have more cognitive resources and thus can process information under cognitive load more efficiently. In addition, HRV reflecting parasympathetic activity correlated with task accuracy. The arousal level estimated using our machine learning model showed its robust relationship with EEG. Despite the limitation of the sample size, the results of this pilot study suggest that the information processing efficiency of the brain under cognitive load is reflected by time-series fluctuations in EEG, which are associated with an individual's task performance. These findings can contribute to the evaluation of the internal state of humans associated with cognitive load and the prediction of human behaviors in various situations under cognitive load.

## 1 Introduction

The total amount of mental activity applied to working memory at a given point in time is called cognitive load (Barrouillet et al., 2007). Cognitive load is an important factor for various activities in daily life, learning, and employment. Cognitive load can easily become excessive because human working memory has limitations in processing capacity and retention time (Chen et al., 2018). Another term, mental load, represents the cost of mental operation and the constraints that are imposed by these costs on performance in cognitive and behavioral tasks (Gopher, 2013). Mental load is a broader and more general concept covering most mental operations, including perception, cognition, and attention, and is thus sometimes linked to fatigue (Díaz-García et al., 2021). We define cognitive load in this study as a more specific load of processing and computation within working memory. Thus, cognitive load might affect task performance but is not necessarily linked to fatigue when the duration of the task is not very long.

In psychology, cognitive load has conventionally been examined using behavioral measures in tasks that are considered to reflect the function of working memory, such as *n*-back tasks (He et al., 2019; Nikolin et al., 2021). However, to accurately assess cognitive load within individuals and to examine the characteristics of information processing associated with cognitive load and its individual differences in more detail, it is necessary to assess the brain activity that underlies working memory.

For this purpose, studies on cognitive load have been conducted using methods to measure brain activity such as electroencephalogram (EEG) (Antonenko et al., 2010; Das et al., 2014) and functional magnetic resonance imaging (fMRI) (Van Dillen et al., 2009; Howard et al., 2015). In EEG, the average powers of alpha (Marsella et al., 2017) and theta (Dan and Reiner, 2017) frequency bands have been used as indices of cognitive load; however, in previous studies, changes in brain activity have not been analyzed over time and those studies were limited in their capability to estimate cognitive load.

Additionally, peripheral physiological responses of the body, especially autonomic nervous system activity such as cardiac activity, can reflect cognitive load. Specifically, it has been reported that heart rate (Cranford et al., 2014) and heart rate variability (HRV) (Solhjoo et al., 2019) positively correlate with cognitive load. Electrodermal activity (EDA) (Nourbakhsh et al., 2012; Vanneste et al., 2021) also responded to cognitive load. However, the relationship between the activities of the autonomic nervous system and brain, especially the functional significance of autonomic nervous activity in the modulation of brain activity accompanying cognitive load, has not been clarified.

On the basis of the findings mentioned above, in this article, we propose a new feature reflecting time-series changes in the power of EEG and examine the relationships of brain and autonomic nervous activities using heart rate and spontaneous skin conductance responses (SCRs) as physiological indices, respectively, with behavioral performance in an *n*-back task under different cognitive loads. Although several types of infra-slow fluctuations in EEG have been reported to date (Kropotov, 2022), we specifically focus on low-frequency fluctuations of EEG power in the alpha frequency band as a candidate new feature of cognitive load (see Section 2.3). This is based on our speculation from previous reports showing that spontaneous infra-slow fluctuations of blood oxygen level-dependent (BOLD) signal in brain regions within the default mode network and task-positive networks measured by fMRI reflect cognitive performance (Bianciardi et al., 2009; Padmala and Pessoa, 2010; Han et al., 2011; Shine et al., 2016), including the performance in the tasks in which cognitive load was manipulated (Vermeij et al., 2014). Recent studies showed that slow fluctuations of brain activity can be detected from EEG signals (Monto et al., 2008; Sato and Katori, 2019), which might correspond to BOLD-fMRI signals. Our speculation is also based on a recent argument that oscillatory activity of the brain, at frequencies especially centered on the alpha band, plays important roles in the development of various cognitive functions in relation to bodily states (Engelen et al., 2023). Furthermore, we applied arousal levels estimated by a machine learning model that we previously developed (Sazuka et al., 2020) to the analysis of data on new EEG features and autonomic activity indices (Sazuka et al., 2021). Through these investigations in this study, we aim to explore the effects of cognitive load experimentally manipulated by the *n*-back task on the characteristics of the infra-slow fluctuations of EEG and autonomic responses. We also aim to examine whether such patterns of brain and body activities can predict the performance of the task under cognitive load. As described above, related previous findings are still limited; thus, this study is a pilot study, which is exploratory rather than hypothesis driven.

## 2 Method

### 2.1 Study overview

This study was conducted as an exploratory pilot study to examine characteristics of EEG and autonomic responses in a situation of cognitive load and to develop a method to estimate the degree of cognitive load using multimodal indices. Data were acquired under a high-cognitive-load condition and a low-cognitive-load condition (control) in *n*-back tasks. We compared the indices of EEG and autonomic responses with task performance between the two conditions. We then ran correlation analyses between the EEG, autonomic, and task performance indices.

### 2.2 Experimental design

#### 2.2.1 Participants

A total of 12 healthy undergraduates participated in this study (eight male and 4 female, age *M* = 20.7, SD = 1.9). Data on EDA from three participants were excluded from analyses owing to technical problems in measurement.

#### 2.2.2 Procedure

The participants conducted a three-back task as the high-cognitive-load condition and a zero-back task as the low-cognitive-load condition.

After obtaining written informed consent to participate in the experiment, devices for measuring EEG and physiological responses were attached to the participants. The participants were given instructions about the experimental task, with a minimal number of trials for training to understand the task. Following a resting period of 5 min with their eyes open, for the purpose of habituation to the experimental situation, the participants performed the three-back task as the high-cognitive-load condition and the zero-back task as the low-cognitive-load condition for 6 min, each counterbalanced in the order of the condition. Under each condition, a single-digit number was randomly and visually presented on a computer display at 3-s intervals consisting of 200 trials of answers for each task. In the three-back task, the participants judged whether the current number was the same as the number presented three trials earlier, whereas in the zero-back task, they judged whether the number presented was even or odd. We produced the program for the *n*-back task by using the Psychtoolbox of Matlab (ver. 3.0.10), and the accuracy of the task (rate of correct answers) and reaction time (of both correct and incorrect answers with temporal resolution of 1 millisecond) were measured by this program. The average of reaction times in all trials under each task was calculated for the index. The program was run, and stimuli were presented to the participants using a PC.

After completing the tasks, we thanked the participants, and they were fully debriefed about the aims of the study. The participants were given 2,000 Japanese Yen (~14.00 USD) for their participation in the experiments. This study was approved by the ethics committee of Nagoya University (NUPSY-2016-A004).

#### 2.2.3 Data acquisition

EEG and physiological responses were measured both in the resting and task periods. The EEG and physiological data measured in the first 60 s of the tasks were excluded from analyses because of the instability of measurements immediately after the initiation of the tasks. Behavioral data were taken during the task period both under the high- and low-cognitive-load conditions; specifically, we measured the accuracy of the *n*-back task (rates of correct responses in the task) and reaction time in the task as behavioral indices. In addition, the participants subjectively rated their feelings of arousal immediately before and after the *n*-back task under the high- and low-cognitive-load conditions using the visual analog scales (VASs) of arousal [0 (not at all aroused) – 100 (extremely aroused)] to confirm the psychological validity of the experimental manipulation of cognitive load.

### 2.3 Feature extraction

#### 2.3.1 EEG

The EEG signals of the frontal region (AF3, AF4, F3, F4, F7, and F8) were measured using a wearable device (EPOC+, Emotiv Inc., www.emotiv.com) at a sampling rate of 256 Hz (Haar and Faisal, 2020; Hebbar et al., 2021). Measurements were conducted using electrodes of saline-soaked felt pads and consisted of two reference electrodes (common mode sense active electrode and driven right leg) positioned at P3/P4. The resolution of the measured EEG was 14 bits at 1 LSB = 0.51 μV and bandwidth of 0.16–43 Hz with digital notch filters at 50 and 60 Hz.

Data preprocessing and basic feature extraction from the acquired EEG are as follows. To all channels, offset removal and the application of a 4–30 Hz 424-order FIR bandpass filter were conducted. Then, the time series of EEG power was extracted by continuously conducting Welch's power spectral density estimation (segmentation of 1 s without overlap) on time-series segments of EEG (20-s-long windows with 1-s shift sequences). EEG band power features were defined as alpha (8–13 Hz) and theta (4–7 Hz), each of which was normalized at a total power of a 4–30 Hz band, hereafter referred to as the first-order time series of alpha power and theta power.

**Figure 1:**
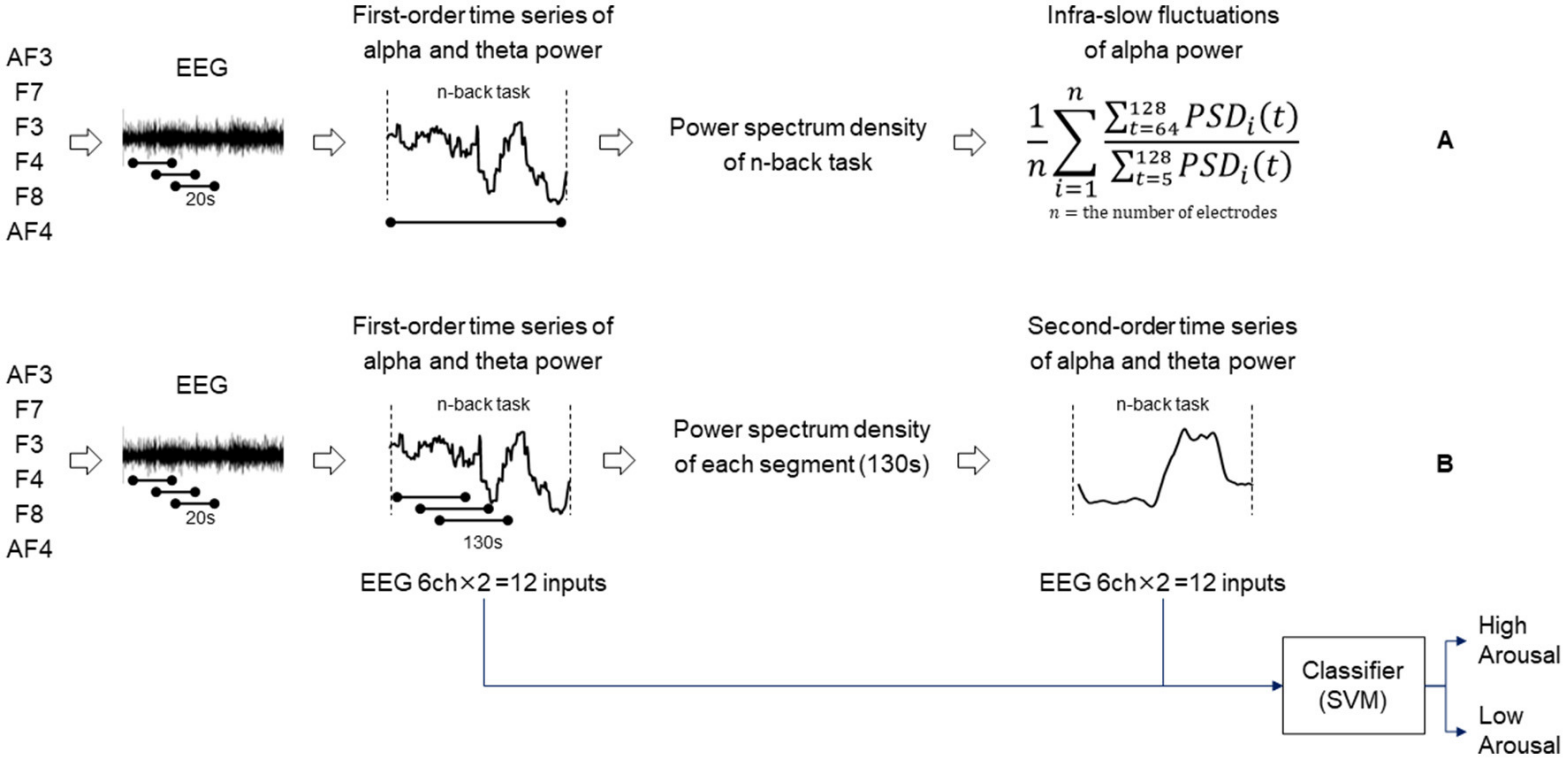
Diagram showing the procedure of extracting infra-slow fluctuations of alpha power **(A)**. Diagram showing the procedure of determining second-order time series of alpha/theta band power and an arousal state estimation model **(B)**.

Then, the features for infra-slow fluctuations of alpha power were extracted. The index of infra-slow fluctuations of alpha power under each task was defined as the power spectrum of fluctuations with a period of 64–128 s normalized by that with a period of 5–128 s computed by Welch's power spectral density estimation method. Infra-slow fluctuations in EEG have been conceptually divided into rhythmic and arrhythmic fluctuations probably with different physiological mechanisms (Kropotov, 2022). We specifically focused on the rhythmic fluctuations of alpha power on the basis of our speculation that the alpha band rhythm is one of the most dominant and salient brain activities and thus can reflect basic levels of processing ability under cognitive load. Then, the average over all channels was used as the index for infra-slow fluctuations of alpha power for the task (Figure 1A).

#### 2.3.2 Physiological responses

Photoplethysmography (PPG) signals for heart rate monitoring from the ring finger and EDA signals for measuring frequencies of SCR from the index and middle fingers of the non-dominant hand were measured using a wearable device (Shimmer3 GSR+, Shimmer Research Inc.) at a sampling rate of 512 Hz. Frequencies of SCR were calculated by Ledalab (Benedek and Kaernbach, 2010). From PPG signals, *R*-waves were detected by a method using noise-reduced pulse signals based on peak detection and autocorrelation methods (Ishikawa et al., 2017). Then, the root mean square of successive differences (RMSSD) of N-N intervals was calculated as HRV.

#### 2.3.3 Arousal state estimation model

The arousal state of each participant was estimated using our previously developed model (Sazuka et al., 2020). This model was trained with data obtained from psychologically controlled experiments using various cognitive tasks in which the participants' arousal was manipulated, using features such as the fluctuations of time series of alpha and theta bands of EEG power. This model was validated to be robust in estimating arousal by using temporarily longer features of alpha power that seem to be valid phenomena as biological responses to cognitive load.

The details of the construction of the classification model are as follows. Using the same feature extraction method explained above, we extracted the first-order time series of alpha and theta powers under various concentration tasks. Additionally, the time series of alpha and theta power features was segmented into windows of 130 s long with 1-s shift sequences to extract the power spectrum of fluctuations with a period of 40–90 s, which is hereafter referred to as the second-order time series of alpha power and theta power (Figure 1B). The combinations of the first- and second-order time series of alpha/theta band powers for the six channels formed 24-dimensional features as input for training a Gaussian kernel support vector machine (SVM) model (Chang and Lin, 2011). The features were normalized using the *z*-transformation method under cognitive tasks. The validation was conducted by applying the leave-one-person-out cross-validation method to prevent overfitting. The model described above was trained using a different *n*-back dataset and has been validated previously (Sazuka et al., 2020). The model was used for estimating arousal levels for this analysis.

### 2.4 Statistical analysis

The difference in each feature between the cognitive tasks was validated by the paired *t*-test following the confirmation of the Kolmogorov–Smirnov normality test and by calculating the effect size *r* defined by the z value divided by the square root of the total number of samples.

The relationship between features was analyzed by calculating the correlation coefficients. In these correlation analyses, the difference values of variables (three-back task minus zero-back task) were used for standardization considering wide individual differences in the variables. Multiple testing correction was conducted by the Benjamini–Hochberg procedure.

## 3 Results

### 3.1 Comparisons between high- and low-cognitive-load conditions

#### 3.1.1 Manipulation check

**Figure 2:**
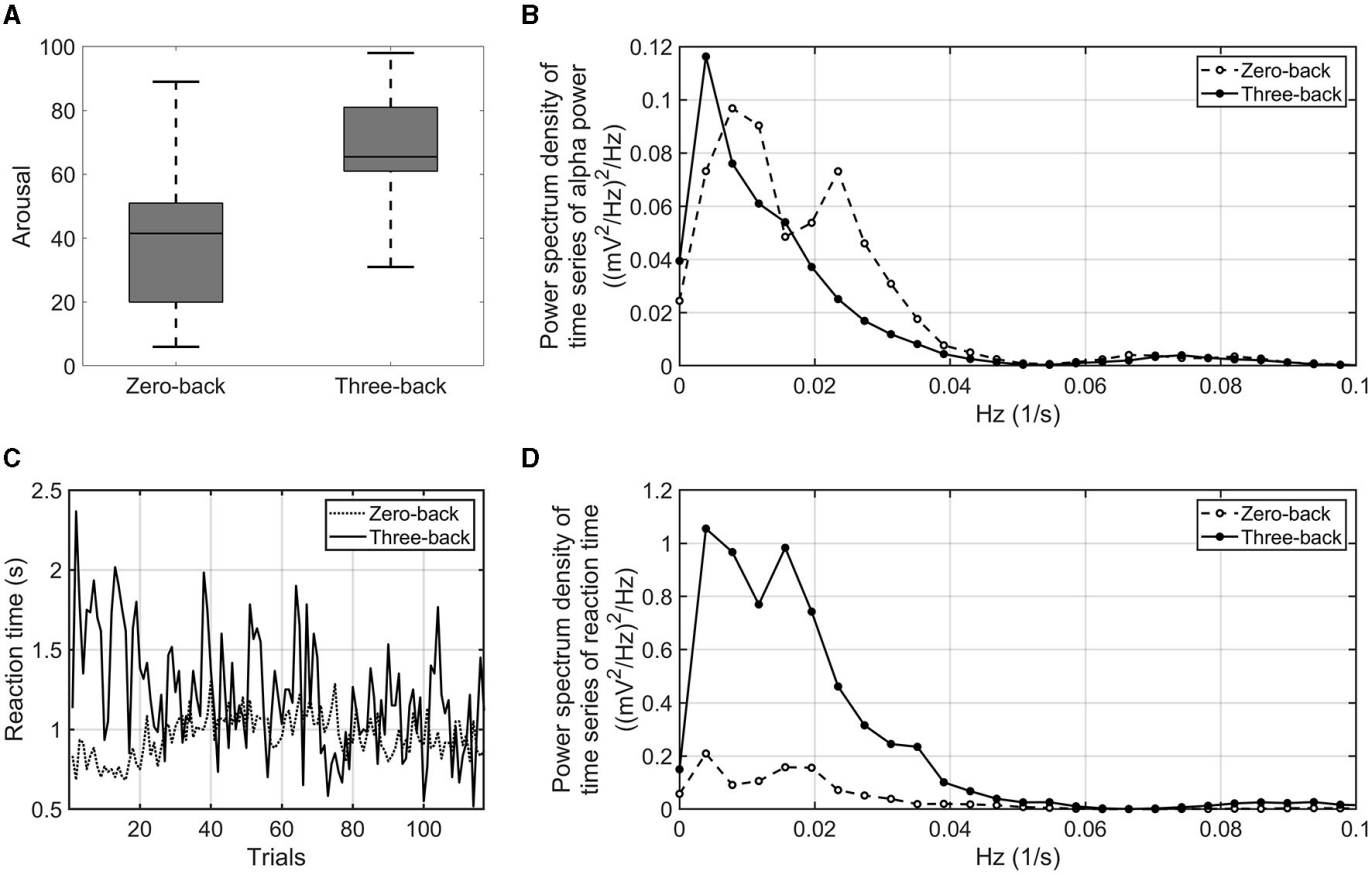
VAS arousal rating of zero-back task and three-back task **(A)**. Power spectrum density of the time series of alpha power averaged over the number of participants and electrodes. The dashed line indicates the power spectrum density of the zero-back task. The solid line indicates the power spectrum density of the three-back task **(B)**. Time series of reaction time in three-back task (solid line) and zero-back task (dashed line) **(C)**. Power spectrum density of the time series of reaction time. The dashed line indicates the power spectrum density in the zero-back task. The solid line indicates the power spectrum density in the three-back task **(D)**.

First, the psychological control of experiments was verified on the basis of subjective evaluation, EEG results, and behavior. As shown in Figure 2A, the VAS rating of arousal was significantly higher in the three-back task than in the zero-back task (*p*-value = 0.016, effect size *r* = 0.749). The alpha power and reaction time during the three-back task were sufficiently smaller and longer (*p*-value = 0.015, effect size *r* = 0.764 and *p*-value = 0.016, effect size *r* = 0.738), respectively, than those in the zero-back task. Thus, it was confirmed that the three-back task relies on an increased cognitive load as compared with the zero-back condition. As the order of tasks (zero-back vs. three-back) was counterbalanced, we examined the effects of the order of the tasks and found none, suggesting few concerns of contamination of human factors such as fatigue and stress (see Supplementary Figure S1).

#### 3.1.2 EEG and behavioral indices

Next, the proposed EEG and behavioral indices were examined as to whether they were sensitive to a difference in high- and low-cognitive-load conditions. As shown in Figure 2B, infra-slow fluctuations of alpha power averaged over the number of participants showed fluctuations in very-low-frequency bands (< 0.1 Hz). The amplitude of alpha power fluctuated with more than a period of 10 s. Peaks of the power spectrum were observed at around 0.01–0.05 Hz under the low-cognitive-load condition; on the other hand, the peaks were attenuated, and the power around 0.01 Hz increased under the high-cognitive-load condition. The infra-slow fluctuations of alpha power in the three-back task were marginally larger than those in the zero-back task (*p*-value = 0.062, effect size *r* = 0.601). For behavioral indices, the interquartile range of reaction time during each task showed a difference between the three-back task and the zero-back task by statistical testing of all the participants' results (*p*-value = 0.002, effect size *r* = 0.852) (Figure 2C). In addition, fluctuations of reaction time among the participants in a very-low-frequency band (< 0.05 Hz) were found to be larger under the high-cognitive-load condition then under the low-cognitive-load condition (*p*-value < 0.001, effect size *r* = 0.900) (Figure 2D). These proposed EEG and behavioral indices, which reflected the cognitive load differences, were used to identify associations between task accuracy and EEG and physiological responses under cognitive load.

### 3.2 Correlations between task accuracy and EEG and physiological responses

#### 3.2.1 Correlation between task accuracy and conventional EEG index

To estimate the cognitive load of participants in *n*-back tasks, task accuracy (which is considered to indicate cognitive performance) was defined as the rate of correct answers for each *n*-back task. The conventional index of cognitive load, which is the average alpha power normalized at a total power of 4–30 Hz bands, did not show a significant correlation with task accuracy, reaction time variability, or the infra-slow fluctuations of alpha power (*r* = 0.147, *p*-value = 0.705; *r* = 0.184, *p*-value = 0.644; and *r* = 0.081, *p*-value = 0.836, respectively). Moreover, the median of the first-order time series of alpha power did not show a significant correlation with task accuracy, reaction time variability, or infra-slow fluctuations of alpha power (*r* = 0.407, *p*-value = 0.262; *r* = 0.029, *p*-value = 0.930; and *r* = 0.276, *p*-value = 0.459, respectively). From the above statistics, both the conventional index of the average alpha power and the first-order feature of alpha power were found not to capture characteristic behaviors under cognitive load. Additionally, the average alpha power was strongly positively correlated with the median of the first-order time series of alpha power (*r* = 0.913, *p*-value < 0.001). For basic data of conventional indices, see Supplementary Tables S1, S2.

#### 3.2.2 Correlation between task accuracy and proposed EEG and physiological indices

**Figure 3:**
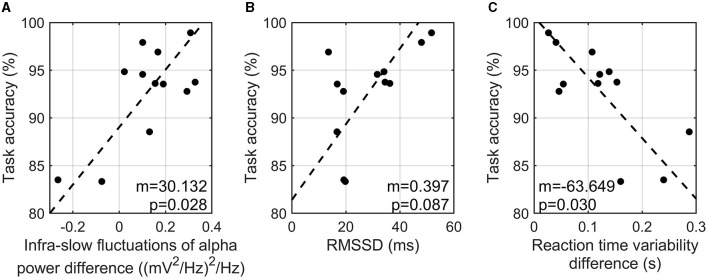
Correlation analysis of task accuracy in three-back task and infra-slow fluctuations of alpha power difference between two tasks **(A)**, RMSSD in three-back task **(B)**, and reaction time variability between two tasks **(C)**.

The difference in the infra-slow fluctuations of alpha power between the high- and low-cognitive-load tasks showed a significant positive correlation with task accuracy (*r* = 0.707, *p*-value = 0.028) (Figure 3A). RMSSD from PPG signals showed a marginal positive correlation with task accuracy (*r* = 0.586, *p*-value = 0.087) (Figure 3B). In addition, the variability of reaction time showed a significant negative correlation with task accuracy (*r* = −0.719, *p*-value = 0.030) (Figure 3C). That is, the participants with a greater enhancement of the infra-slow fluctuations of alpha power exhibited a higher task accuracy, a higher HRV in the three-back task, and a more constant reaction time in the high-cognitive-load task than in the low-cognitive-load task. On the other hand, SCR showed no consistent patterns of correlation with task accuracy (*r* = 0.428, *p*-value = 0.329).

### 3.3 Further investigation of infra-slow fluctuations of alpha power

**Figure 4:**
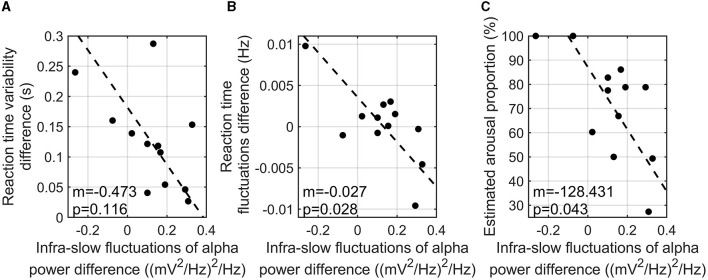
Correlation analysis of reaction time variability (i.e., interquartile reaction time range) difference between three-back and zero-back tasks and infra-slow fluctuations of alpha power difference between two tasks (*m* is the slope of the regression line; *p* is the *p*-value of coefficient) **(A)**. Correlation analysis of reaction time fluctuation and the infra-slow fluctuations of alpha power differences between two tasks (*m* is the slope of the regression line; *p* is the *p*-value of coefficient) **(B)**. Correlation analysis of estimated high arousal portion in three-back task and the infra-slow fluctuations of alpha power between two tasks (*m* is the slope of the regression line; *p* is the *p*-value of coefficient) **(C)**.

Since infra-slow fluctuations of alpha power were found to show a correlation with task accuracy, further investigation of the correlation analyses was conducted. To investigate the relationship between reaction time variability and the infra-slow fluctuations of alpha power, the difference in the interquartile reaction time range between the three-back and zero-back tasks and the difference in the infra-slow fluctuations of alpha power between the two tasks were calculated. As a result, a higher infra-slow EEG alpha power was found to be significantly associated with a shorter reaction time (*r* = −0.817, *p*-value = 0.003). The infra-slow fluctuations of alpha power also tended to correlate with less variability in reaction time (*r* = −0.548, *p*-value = 0.116) (Figure 4A), but not significantly. The index of characteristic fluctuations of reaction time was defined as the mean power spectrum density function and is hereafter referred to as reaction time fluctuations. The difference in reaction time fluctuations between the three-back and zero-back tasks was calculated. We found that reaction time fluctuations showed a significantly negative correlation with the infra-slow fluctuations of alpha power, that is, the participants with a greater enhancement of the infra-slow fluctuations of alpha power presented smaller reaction time fluctuations (correlation = −0.714, *p*-value = 0.028) (Figure 4B).

We estimated the arousal levels of the participants during *n*-back tasks using our SVM model as explained above. The difference in the infra-slow fluctuations of alpha power between the high- and low-cognitive-load tasks was negatively correlated with arousal level, which is the time portion of estimated high arousal during the three-back task (*r* = −0.663, *p*-value = 0.043) (Figure 4C). This finding indicates that the participants with higher infra-slow fluctuations of alpha power did not show an excessive arousal level, which is related to the depletion of cognitive resources during the high-cognitive-load task.

No significant association of the physiological response index was found with the EEG features and estimated arousal levels (see Supplementary Figure S2).

## 4 Discussion

The results of this study indicated that the enhanced infra-slow fluctuations of EEG alpha power during the *n*-back tasks correlated with better task performance. These findings suggest that the infra-slow fluctuations of EEG alpha power might be linked with efficient processing within working memory when cognitive load increases.

Although the phenomenon of infra-slow fluctuations of EEG including the alpha band is already known, its significance in cognitive ability has not been clarified. It has been reported that the phase of infra-slow fluctuation of the full-band EEG correlated with accuracy in perceptual tasks such as somatosensory stimulus detection, suggesting that the infra-slow fluctuations of EEG reflect the excitability dynamics of cortical networks (Monto et al., 2008). Another study indicated that the infra-slow fluctuations of EEG, mainly in alpha power, positively correlated with reaction time in simpler visual and auditory stimulus discrimination tasks, suggesting that larger infra-slow fluctuations of EEG are associated with lower performance in the tasks (Sato and Katori, 2019). This finding is seemingly the opposite of our finding indicating the association between larger infra-slow fluctuations of alpha power and superior task performance. One possible interpretation of these contradictory findings is to consider the speculation that the relationship between infra-slow fluctuations of EEG and task performance depends on the complexity and difficulty of the task, and the relationship can be reversed depending on the characteristics of the tasks. The validity of this speculation should be clarified in future studies. As Sato and Katori (2019) reasoned, these findings might correspond to previous findings from fMRI studies showing that the low-frequency oscillations in BOLD signal fluctuations in large-scale neural networks such as the default-mode network were linked to the performance in some cognitive tasks (Bianciardi et al., 2009; Padmala and Pessoa, 2010; Han et al., 2011; Hiltunen et al., 2014; Shine et al., 2016), including the tasks in which the cognitive load was manipulated (Vermeij et al., 2014). However, such previous studies (Monto et al., 2008; Sato and Katori, 2019) relied on the conventional frequency analysis of EEG power. Note that the conventional index (average alpha power) was not sensitive to cognitive load in the present study but only the infra-slow fluctuations of alpha power, which we proposed as a new feature responding to behavioral indices under cognitive load. This might explain the differences between the findings of previous studies and our study. Our study also suggests the potential utility of the new feature of the infra-slow alpha fluctuations, which we propose here, even though this is still a pilot study.

The mechanisms underlying the infra-slow fluctuations of alpha power observed in this study are not clear yet. Kropotov (2022) pointed out the significance of 0.1 Hz oscillations in arterial blood pressure coupled with fluctuations of efferent sympathetic nervous activity (Julien, 2006) in the rhythmic spontaneous infra-slow fluctuations of EEG. One possibility is that such cardiovascular activity might play important roles in shaping, maintaining, and enhancing EEG fluctuations, especially in the alpha band, through cerebral vasomotion (Nikulin et al., 2014) and/or the physical principle of the phase–amplitude coupling (Klimesch, 2018).

The mechanisms underlying the correlation between the infra-slow fluctuations of alpha power and the performance of cognitive tasks are also unclarified. We observed that the variance of reaction time increased under the high-cognitive-load condition compared with that under the low-cognitive-load condition. To the best of our knowledge, this phenomenon itself is a new finding, presumably reflecting a type of clogging of cognitive processing caused by cognitive load and the shortage of working memory resources. In previous studies using *n*-back tasks, reaction time was examined as the average over all trials in the task; thus, temporal fluctuations of reaction time have not been addressed. We observed a tendency of negative correlations between the infra-slow fluctuations of alpha power and the frequencies of reaction time fluctuations. This finding suggests that our new EEG feature could capture the characteristics of behaviors that might be related to their hidden mechanisms attributable to the cognitive load.

The arousal levels estimated by our previously constructed SVM model showed a negative association with the infra-slow fluctuations of alpha power. This suggests that the brain is not overloaded cognitively and can process a load efficiently. The strengths of our model are 2-fold. First, we built our model on the basis of characteristic biological responses that might reflect arousal levels. Specifically, our model uses the temporarily longer feature of alpha power, a phenomenon that seems to be characteristic of cognitive load, as the feature quantity. Most of the previous studies (Lawhern et al., 2018; Salama et al., 2018; Fahimi et al., 2019) are based on engineering interests and do not pay much attention to whether they can capture valid responses in biological phenomena. For example, many previous models were constructed by analyzing EEG in a time window of < 10 s. It is expected that even such models can capture some characteristics of input data such as EEG in that short time window. However, no matter how excellent the calculations of the machine learning classifiers are, if the phenomenon of interest, such as arousal level, is reflected in biological responses that are temporarily longer than the time window, it cannot be captured by the model in principle. Second, we obtained high-quality data in a precisely controlled psychological experiment. No matter how much data are available, if their quality is poor, a well-tuned model cannot be constructed. Compared with computer science, in psychology, one attempts to strictly define the target phenomenon, logically draws hypotheses, and empirically tests the hypotheses through elaborately controlled human experiments. For biological data, especially for the evaluation of the internal state of humans similarly to this study, which is difficult to measure in large amounts owing to the nature of experiments, it is important to control well the measurements of even small amounts of biological data.

Here, note that EDA and HRV showed no significant correlations with the arousal level estimated using the model. These results suggest that the arousal levels estimated by our model are not direct representations of peripheral physiological activity reflected by EDA and HRV but might reflect more integrated and abstract forms of representations of arousal constructed in the brain.

This is a pilot study conducted with a small sample size and a constraint of gender imbalance. This is a limitation of this study that is common to previous studies (Monto et al., 2008; Hiltunen et al., 2014; Hebbar et al., 2021); thus, caution is needed when interpreting the results. However, even taking such limitations into account, the results of this study suggest that the efficiency of brain information processing under cognitive load is reflected by temporal fluctuations in alpha EEG bands, which are associated with individual task performance. Arousal levels, marginally reflected in HRV (a parasympathetic index) and estimated by a machine learning model, might be associated with individual task performance. This finding can contribute to the assessment of the internal state of humans under cognitive load and to the prediction of behaviors. In addition, the devices used in this study to measure EEG and physiological responses are wearable, less expensive, and simpler than conventional neuroimaging methods such as positron emission tomography and fMRI. The findings obtained in this study and the model developed on the basis of simpler methods should be beneficial for future social implementation.

## Data availability statement

The datasets presented in this article are not readily available because we are afraid that we could not publicize our data, since we have a commercial enterprise in authors. Requests to access the datasets should be directed to NS, naoya.sazuka@sony.com.

## Ethics statement

The studies involving humans were approved by the Ethical Committee of Nagoya University. The studies were conducted in accordance with the local legislation and institutional requirements. The participants provided their written informed consent to participate in this study.

## Author contributions

NS: Conceptualization, Formal analysis, Investigation, Methodology, Project administration, Supervision, Validation, Visualization, Writing—original draft, Writing—review & editing. KK: Data curation, Formal analysis, Investigation, Methodology, Validation, Visualization, Writing—original draft, Writing—review & editing. YK: Funding acquisition, Project administration, Supervision, Writing—review & editing, Investigation. TO: Data curation, Investigation, Methodology, Resources, Validation, Visualization, Writing—review & editing. HO: Conceptualization, Investigation, Methodology, Project administration, Resources, Supervision, Validation, Writing—original draft, Writing—review & editing.

## References

[B1] Antonenko P. Paas F. Grabner R. Van Gog T. (2010). Using electroencephalography to measure cognitive load. Educ. Psychol. Rev. 22, 425–438. 10.1007/s10648-010-9130-y

[B2] Barrouillet P. Bernardin S. Portrat S. Vergauwe E. Camos V. (2007). Time and cognitive load in working memory. J. Exp. Psychol. 33, 570–585. 10.1037/0278-7393.33.3.570 17470006

[B3] Benedek M. Kaernbach C. (2010). Decomposition of skin conductance data by means of nonnegative deconvolution. Psychophysiology 47, 647–658. 10.1111/j.1469-8986.2009.00972.x 20230512 PMC2904901

[B4] Bianciardi M. Fukunaga M. van Gelderen P. Horovitz S. G. de Zwart J. A. Duyn J. H. (2009). Modulation of spontaneous fMRI activity in human visual cortex by behavioral state. Neuroimage 45, 160–168. 10.1016/j.neuroimage.2008.10.034 19028588 PMC2704889

[B5] Chang C.-C. Lin C.-J. (2011). LIBSVM: a library for support vector machines. ACM Transact. Intell. Syst. Technol. 2, 1–27 10.1145/1961189.1961199

[B6] Chen O. Castro-Alonso J. C. Paas F. Sweller J. (2018). Extending cognitive load theory to incorporate working memory resource depletion: evidence from the spacing effect. Educ. Psychol. Rev. 30, 483–501. 10.1007/s10648-017-9426-2

[B7] Cranford K. N. Tiettmeyer J. M. Chuprinko B. C. Jordan S. Grove N. P. (2014). Measuring load on working memory: the use of heart rate as a means of measuring chemistry students' cognitive load. J. Chem. Educ. 91, 641–647. 10.1021/ed400576n

[B8] Dan A. Reiner M. (2017). EEG-based cognitive load of processing events in 3D virtual worlds is lower than processing events in 2D displays. Int. J. Psychophysiol. 122, 75–84. 10.1016/j.ijpsycho.2016.08.013 27592084

[B9] Das R. Chatterjee D. Das D. Sinharay A. Sinha A. (2014). “Cognitive load measurement-a methodology to compare low cost commercial eeg devices,” in 2014 International Conference on Advances in Computing, Communications and Informatics (ICACCI) (IEEE), 1188–1194.

[B10] Díaz-García J. González-Ponce I. Ponce-Bordón J. C. López-Gajardo M. Á. Ramírez-Bravo I. Rubio-Morales A.. (2021). Mental load and fatigue assessment instruments: a systematic review. Int. J. Environ. Res. Public Health 19:419. 10.3390/ijerph19010419 35010678 PMC8744873

[B11] Engelen T. Solcà M. Tallon-Baudry C. (2023). Interoceptive rhythms in the brain. Nat. Neurosci. 26, 1670–1684. 10.1038/s41593-023-01425-1 37697110

[B12] Fahimi F. Zhang Z. Goh W. B. Lee T. S. Ang K. K. Guan C. (2019). Inter-subject transfer learning with an end-to-end deep convolutional neural network for EEG-based BCI. J. Neural Eng. 16:026007. 10.1088/1741-2552/aaf3f6 30524056

[B13] Gopher D. (2013). “Analysis and measurement of mental load,” in International Perspectives on Psychological Science, II: The State of the Art, eds P. Bertelson, P. Eelen, and G. d'Ydewalle (London: Psychology Press), 265–91.

[B14] Haar S. Faisal A. A. (2020). Brain activity reveals multiple motor-learning mechanisms in a real-world task. Front. Hum. Neurosci. 14:354. 10.3389/fnhum.2020.00354 32982707 PMC7492608

[B15] Han Y. Wang J. Zhao Z. Min B. Lu J. Li K.. (2011). Frequency-dependent changes in the amplitude of low-frequency fluctuations in amnestic mild cognitive impairment: a resting-state fMRI study. Neuroimage 55, 287–295. 10.1016/j.neuroimage.2010.11.059 21118724

[B16] He D. Donmez B. Liu C. C. Plataniotis K. N. (2019). High cognitive load assessment in drivers through wireless electroencephalography and the validation of a modified N-back task. IEEE Transact. Hum. Mach. Syst. 49, 362–371. 10.1109/THMS.2019.2917194

[B17] Hebbar P. A. Bhattacharya K. Prabhakar G. Pashilkar A. A. Biswas P. (2021). Correlation between physiological and performance-based metrics to estimate pilots' cognitive workload. Front. Psychol. 12:555446. 10.3389/fpsyg.2021.555446 33959060 PMC8093450

[B18] Hiltunen T. Kantola J. Abou Elseoud A. Lepola P. Suominen K. Starck T.. (2014). Infra-slow EEG fluctuations are correlated with resting-state network dynamics in fMRI. J. Neurosci. 34, 356–362. 10.1523/JNEUROSCI.0276-13.2014 24403137 PMC6608153

[B19] Howard S. J. Burianová H. Ehrich J. Kervin L. Calleia A. Barkus E.. (2015). Behavioral and fMRI evidence of the differing cognitive load of domain-specific assessments. Neuroscience 297, 38–46. 10.1016/j.neuroscience.2015.03.047 25818553

[B20] Ishikawa T. Hyodo Y. Miyashita K. Yoshifuji K. Komoriya Y. Imai Y. (2017). “Wearable motion tolerant ppg sensor for instant heart rate in daily activity,” in International Conference on Bio-Inspired Systems and Signal Processing, Vol. 5 (Porto: SCITE Press), 126–133.

[B21] Julien C. (2006). The enigma of Mayer waves: facts and models. Cardiovasc. Res. 70, 12–21. 10.1016/j.cardiores.2005.11.008 16360130

[B22] Klimesch W. (2018). The frequency architecture of brain and brain body oscillations: an analysis. Eur. J. Neurosci. 48, 2431–2453. 10.1111/ejn.14192 30281858 PMC6668003

[B23] Kropotov J. D. (2022). The enigma of infra-slow fluctuations in the human EEG. Front. Hum. Neurosci. 16:928410. 10.3389/fnhum.2022.928410 35982689 PMC9378968

[B24] Lawhern V. J. Solon A. J. Waytowich N. R. Gordon S. M. Hung C. P. Lance B. J. (2018). EEGNet: a compact convolutional neural network for EEG-based brain–computer interfaces. J. Neural Eng. 15:056013. 10.1088/1741-2552/aace8c 29932424

[B25] Marsella P. Scorpecci A. Cartocci G. Giannantonio S. Maglione A. G. Venuti I.. (2017). EEG activity as an objective measure of cognitive load during effortful listening: A study on pediatric subjects with bilateral, asymmetric sensorineural hearing loss. Int. J. Pediatr. Otorhinolaryngol. 99, 1–7. 10.1016/j.ijporl.2017.05.006 28688548

[B26] Monto S. Palva S. Voipio J. Palva J. M. (2008). Very slow EEG fluctuations predict the dynamics of stimulus detection and oscillation amplitudes in humans. J. Neurosci. 28, 8268–8272. 10.1523/JNEUROSCI.1910-08.2008 18701689 PMC6670577

[B27] Nikolin S. Tan Y. Y. Schwaab A. Moffa A. Loo C. K. Martin D. (2021). An investigation of working memory deficits in depression using the n-back task: a systematic review and meta-analysis. J. Affect. Disord. 284, 1–8. 10.1016/j.jad.2021.01.084 33581489

[B28] Nikulin V. V. Fedele T. Mehnert J. Lipp A. Noack C. Steinbrink J.. (2014). Monochromatic ultra-slow (~0.1 Hz) oscillations in the human electroencephalogram and their relation to hemodynamics. Neuroimage 97, 71–80. 10.1016/j.neuroimage.2014.04.008 24732648

[B29] Nourbakhsh N. Wang Y. Chen F. Calvo R. A. (2012). “Using galvanic skin response for cognitive load measurement in arithmetic and reading tasks,” in Proceedings of the 24th Australian Computer-Human Interaction Conference (New York, NY), 420–423.

[B30] Padmala S. Pessoa L. (2010). Moment-to-moment fluctuations in fMRI amplitude and interregion coupling are predictive of inhibitory performance. Cogn. Affect. Behav. Neurosci. 10, 279–297. 10.3758/CABN.10.2.279 20498351 PMC2946362

[B31] Salama E. S. El-Khoribi R. A. Shoman M. E. Shalaby M. A. W. (2018). EEG-based emotion recognition using 3D convolutional neural networks. Int. J. Adv. Comp. Sci. Appl. 9, 329–337. 10.14569/IJACSA.2018.09084332845859

[B32] Sato N. Katori Y. (2019). “Infra-slow electroencephalogram power associates with reaction time in simple discrimination tasks,” in International Conference on Neural Information Processing (Cham: Springer), 501–511.

[B33] Sazuka N. Katsumata K. Komoriya T. Oba T. Ohira T. (2021). “Slow EEG fluctuation reflects cognitive load and affective arousal,” in Annual Conference of the Society for Affective Science (Virtual).

[B34] Sazuka N. Komoriya Y. Ezaki T. Oba T. Ohira T. (2020). “Human affective states estimation by a model of meta-level patterns of EEG,” in Annual Conference of the Society for Affective Science (Virtual).

[B35] Shine J. M. Bissett P. G. Bell P. T. Koyejo O. Balsters J. H. Gorgolewski K. J.. (2016). The dynamics of functional brain networks: integrated network states during cognitive task performance. Neuron 92, 544–554. 10.1016/j.neuron.2016.09.018 27693256 PMC5073034

[B36] Solhjoo S. Haigney M. C. McBee E. van Merrienboer J. J. Schuwirth L. Artino A. R.. (2019). Heart rate and heart rate variability correlate with clinical reasoning performance and self-reported measures of cognitive load. Sci. Rep. 9, 1–9. 10.1038/s41598-019-50280-3 31604964 PMC6789096

[B37] Van Dillen L. F. Heslenfeld D. J. Koole S. L. (2009). Tuning down the emotional brain: an fMRI study of the effects of cognitive load on the processing of affective images. Neuroimage 45, 1212–1219. 10.1016/j.neuroimage.2009.01.016 19349235

[B38] Vanneste P. Raes A. Morton J. Bombeke K. Van Acker B. B. Larmuseau C.. (2021). Towards measuring cognitive load through multimodal physiological data. Cognit. Technol. Work 23, 567–585. 10.1007/s10111-020-00641-0

[B39] Vermeij A. Meel-van den Abeelen A. S. Kessels R. P. van Beek A. H. Claassen J. A. (2014). Very-low-frequency oscillations of cerebral hemodynamics and blood pressure are affected by aging and cognitive load. Neuroimage 85, 608–615. 10.1016/j.neuroimage.2013.04.107 23660026

