# Supplementary material for: Association of brain–autonomic activities and task accuracy under cognitive load: a pilot study using electroencephalogram, autonomic activity measurements, and arousal level estimated by machine learning
Source: Front Hum Neurosci. 2024 Feb 29;18:1272121. doi: 10.3389/fnhum.2024.1272121 (PMC10937530; doi:10.3389/fnhum.2024.1272121)
Supplement: Supplementary file 1 [file Data_Sheet_1.pdf]

Supplementary Material

1 Supplementary Figures and Tables

1.1 Manipulation check for task order effect on task accuracy

To analyze effects of the task order on task accuracy, we examined the difference of performance index between the order of the task conducted, since the task order was counterbalanced between participants. We found that the task accuracy was identical between the two tasks which indicates the order of tasks does not have significant influence on performance (Supplementary Figure S1). This observation was supported by statistical analyses, which did not reveal any significant differences in task accuracy between the order of the task (p-value=0.977). This result suggests that effects of human factors such as fatigue and stress accompanying conductance of the task on the task accuracy were small.

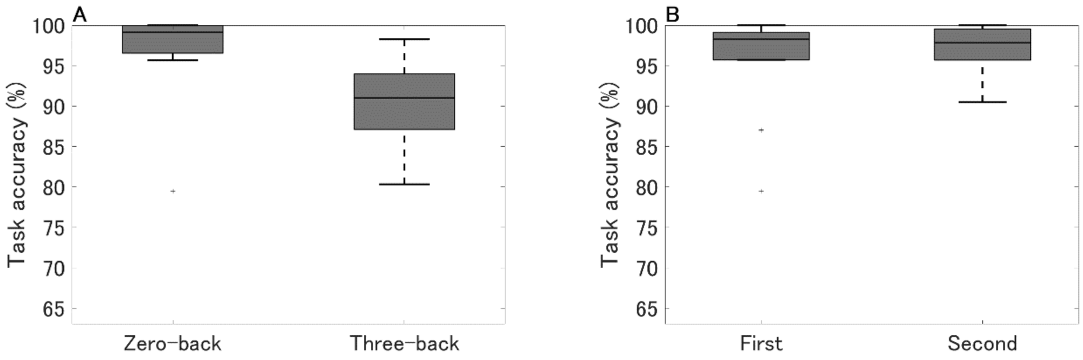

Supplementary Figure S1. The figure shows the task accuracy of the participants, by task type (zero-back and three-back) (A) and the order of task (first and second task) (B).

1.2 Correlation between conventional indices

We performed a statistical analysis to examine the difference between zero-back and three-back tasks in conventional features (Supplementary Table S1 and S2). Regarding the analyses of alpha and theta power, we found significant differences between the two tasks. However, when examining the correlations between these EEG measures and performance indicators, such as task accuracy and reaction time variability, we did not observe any significant correlations.

As for the RMSSD we found correlations between RMSSD and task accuracy. However, in the comparison between the zero-back and three-back tasks, we did not find significant differences in RMSSD; suggesting further analysis is needed by acquiring more samples for peripheral features.

Based on these findings, it appears that the alpha and theta power, as well as the RMSSD, may not be directly indicative of cognitive load in the context of our study. While there were significant

differences in these EEG measures between the high and low demanding tasks, they did not show consistent correlations with performance indicators.

Supplementary Table S1. Table of statistical difference between three-back and zero-back tasks by each feature. The statistical tests comparing the alpha and theta power (calculated as median under each task), as well as RMSSD, between the three-back and zero-back tasks.

	**p-Value**	**Effect size (Cohens' D)**
**Theta power**	**0.004**	**1.400**
**Alpha power**	**0.003**	**-1.230**
**RMSSD**	**0.272**	**-0.024**

Supplementary Table S2. Table of correlation between performance index (reaction time and task accuracy) by each feature. statistical tests comparing the alpha and theta power (calculated as median under each task), as well as the root mean square of successive differences (RMSSD), between the three-back and zero-back tasks.

	**Reaction time**		**Task accuracy**	
	**Correlation coefficient (r)**	**p-value**	**Correlation coefficient (r)**	**p-value**
**Theta power**	**-0.479**	**0.115**	**-0.165**	**0.609**
**Alpha power**	**0.109**	**0.735**	**0.289**	**0.362**
**RMSSD**	**-0.379**	**0.223**	**0.586**	**0.045**

1.3 Further investigation on physiological responses

RMSSD and SCR were calculated from PPG and EDA signals as indices of the parasympathetic and sympathetic activities, respectively. PPG and EDA signals were segmented into 40-second-long windows with 1-second shift sequences. RMSSD and SCR were calculated for each window and their medians during each cognitive task were calculated. Normalized SCR frequency, which is the SCR in the three-back task divided by the SCR in the baseline task, was calculated owing to large individual differences in SCR. No significant association of RMSSD and SCR with the infra-slow fluctuations of alpha power was found (r=0.290, p-value=0.451 and r=0.522, p-value=0.233 respectively). RMSSD and frequencies of SCR also showed no significant correlations with arousal level (r=-0.540, p-value=0.116 and r=-0.493, p-value=0.261 respectively). Nevertheless, visual

inspection suggests that participants with higher HRV and higher SCR during the high-cognitive-load task showed a low arousal level during the high-cognitive-load task (Supplementary Figure S2).

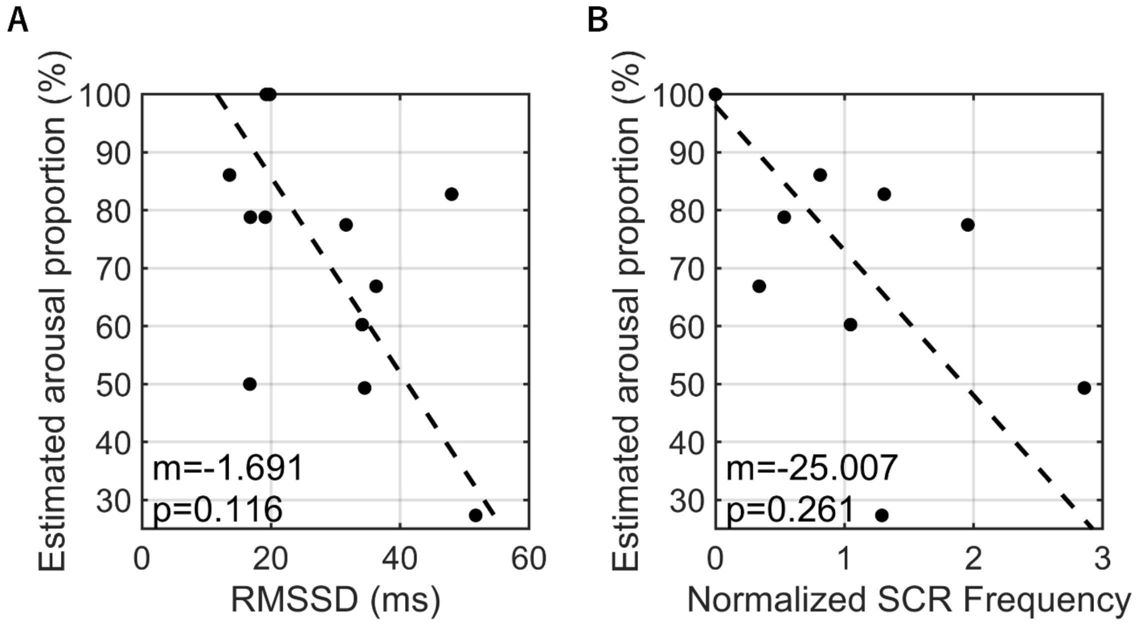

Supplementary Figure S2. Correlation analysis of estimated high arousal portion in three-back task and RMSSD (A), and normalized SCR frequency (B). m is the slope of the regression line; p is the p-value of coefficient.

Parasympathetic (vagal) nervous system activity, as reflected by HRV (RMSSD) during a task, was marginally correlated to task accuracy, but not to the infra-slow fluctuations of alpha power. Sympathetic nervous system activity, as reflected by spontaneous SCR frequency, showed no correlations to either task performance or brain activity. These findings suggest that brain activity reflected by the infra-slow fluctuations of alpha power can affect the performance of cognitive tasks independently from autonomic physiological responses. The involvement of HRV in task performance is difficult to explain. However, a meta-analysis of neuroimaging studies indicated that HRV correlates with activity levels in the medial prefrontal and anterior cingulate cortices, the amygdala, and the striatum of the brain (Thayer et al., 2012). Thus, one possibility is that HRV can indirectly reflect cognitive ability and the processing efficacy of the brain.

2 References

Thayer, J. F., Åhs, F., Fredrikson, M., Sollers III, J. J., and Wager, T. D. (2012). A meta-analysis of heart rate variability and neuroimaging studies: implications for heart rate variability as a marker of stress and health. Neuroscience & Biobehavioral Reviews, 36(2), 747–756. doi.org/10.1016/j.neubiorev.2011.11.009
